# Emergency Medical Responses at US Immigration and Customs Enforcement Detention Centers in California

**DOI:** 10.1001/jamanetworkopen.2023.45540

**Published:** 2023-11-29

**Authors:** Annette M. Dekker, Jennifer Farah, Parveen Parmar, Atilla B. Uner, David L. Schriger

**Affiliations:** 1 Department of Emergency Medicine, David Geffen School of Medicine, University of California, Los Angeles; 2 Department of Emergency Medicine, University of California, San Diego; 3 Department of Emergency Medicine, Keck School of Medicine, University of Southern California, Los Angeles

## Abstract

**Importance:**

Knowledge of medical care in US Immigration and Customs Enforcement (ICE) detention centers is limited. Reviews show high rates of preventable deaths while in detention due to substandard medical care within detention centers.

**Objective:**

To describe characteristics of medical emergencies at ICE detention centers in California.

**Design, Setting, and Participants:**

This cross-sectional study analyzed emergency medical services (EMS)–reported medical emergencies activated through 911 from January 1, 2018, to December 31, 2022, at 3 of the 5 ICE detention centers in California: Adelanto ICE Processing Center, Imperial Regional Detention Facility, and Otay Mesa Detention Center.

**Exposure:**

Medical services requiring 911 activation.

**Main Outcomes and Measures:**

Data included the number of EMS-reported medical emergencies; ICE-reported medical emergencies; patient age and sex; and medical data in the EMS report, including primary symptom, EMS provider (emergency medical technician or paramedic) impression, vital signs, and interventions performed, using descriptive statistics.

**Results:**

In this analysis of 3 detention centers with a mean daily census of 775 (range, 504-1001) individuals per center, there was a median of 68 (IQR, 10-88) EMS-reported emergencies per center per year for a total of 1224 emergencies. The EMS-reported emergencies involved 881 males (72%) and 338 females (28%) with a median age of 39.0 (IQR 30.0-49.0) years. The ratios of female-to-male EMS-reported emergencies were 0.51 (95% CI, 0.31-0.87) at Adelanto, 0.86 (95% CI, 0.31-2.43) at Imperial, and 1.60 (95% CI, 1.40-1.83) at Otay Mesa. Pregnancy-related emergencies accounted for 12.4% (42 of 338) of emergencies in females. A total of 357 individuals (29.2%) had at least 1 abnormal vital sign during their EMS encounter. Psychiatric emergencies comprised 48 EMS responses (3.9%), despite higher rates of ICE-reported psychiatric-related complaints. The median number of monthly EMS-reported emergencies across all 3 centers was 3 (IQR, 0-9) vs 4 ICE-reported emergencies (IQR, 1-15).

**Conclusions and Relevance:**

In this study of medical emergencies at 3 of 5 ICE detention facilities in California, there were discrepancies in EMS vs ICE reported emergencies. The data suggested that vulnerable populations, such as pregnant women, had an increased number of medical emergencies, while individuals with psychiatric emergencies accounted for fewer reported emergencies. Based on these findings, emergency medical care provision in ICE detention centers requires increased transparency to ensure appropriate delivery of care.

## Key Points

**Question:**

What were the characteristics of medical emergencies at US Immigration and Customs Enforcement (ICE) detention centers in California from 2018 to 2022?

**Findings:**

In this cross-sectional analysis of 3 detention centers, emergency medical services reported a median of 68 emergencies per center per year for a total of 1224 medical emergencies. The median number of monthly emergency medical services–reported emergencies across all 3 centers was 3, while that of monthly ICE-reported emergencies was 4.

**Meaning:**

These findings suggest a need for an increased understanding of how medical emergencies are managed at ICE detention centers to ensure that the health care system meets the needs of detained individuals.

## Teaser

This cross-sectional study describes emergency medical services– and US Immigration and Customs Enforcement (ICE)–reported medical emergencies in California detention centers.

## Introduction

There have been longstanding concerns regarding substandard health care in US Immigration and Customs Enforcement (ICE) detention facilities, with recent attention focused on deaths of individuals held in detention.^1,2,3^ Deaths in ICE facilities typically occur in young individuals (mean age, 42.7 years) with few comorbidities.^4^ One-half of the fatalities are due to potentially preventable causes, such as COVID-19 and suicide.^5^ Systematic substandard care has been identified as a factor associated with these deaths, including lack of recognition of severe illness, medical staff dismissal of concerns about individuals’ health, and delays in activating external emergency care. These findings suggest that there are near misses not captured in death reviews.^6^

Most US health systems have mechanisms to review poor outcomes. However, oversight of medical care in ICE detention centers is limited. Nationally, ICE Enforcement and Removal Operations contracts with the Nakamoto Group Inc to conduct annual facility inspections, while the Office of Detention Oversight inspects facilities every 3 years. Inspections evaluate facility compliance with Performance-Based National Detention Standards.^7^ A recent report by the Department of Homeland Security (DHS) Office of Inspect General stated that Nakamoto’s inspections “do not fully examine actual conditions or identify all compliance deficiencies,”^7^^(p4)^ while Office of Detention Oversight inspections are “too infrequent to ensure the facilities implement all corrections.”^7^^(p4)^ Moreover, facilities are not held accountable for correcting deficiencies.^7^ Mandated medical reviews occur for individuals who die while in ICE custody; however, no systematic reviews currently exist to monitor other outcomes.^8^ In California, legislators have increased oversight of ICE detention centers by passing assembly bill No. 103, which establishes attorney general oversight of detention facilities,^9^ and senate bill No. 29, which prohibits local governments from expanding or entering into contracts with the federal government or private companies for immigration detention.^10^

The aim of this study is to expand our current understanding of medical emergencies in ICE detention centers in California. The study explores rates and characteristics of emergency medical services (EMS)–reported emergencies as well as ICE-reported medical emergencies for individuals held in detention centers in California.

## Methods

This cross-sectional analysis examined medical emergency responses at ICE detention centers in California from January 1, 2018, to December 31, 2022. The study was deemed exempt by the University of California, Los Angeles institutional review board, with a waiver of informed consent given that deidentified and publicly available data were used. Data were obtained and triangulated across 3 sources, including DHS ICE–mandated reporting, California Department of Justice (CA DOJ) annual inspections, and EMS agencies. This study followed the Strengthening the Reporting of Observational Studies in Epidemiology (STROBE) reporting guideline.^11^

### DHS ICE

Dates of operation, mean daily population, and number of onsite medical personnel were obtained via ICE-authorized facility list reports available on the DHS ICE website.^12,13^ Data not posted on DHS ICE website were extracted using an internet archive, Wayback Machine.^14^ Noncumulative monthly mean daily populations were calculated from ICE-reported cumulative mean daily populations. Data were available from July 1, 2018, to September 30, 2022.

Additional information was obtained via ICE Enforcement and Removal Operations facility inspections available on the DHS ICE website.^15^ Facility inspections included ICE self-reported medical incidents per month from January 1, 2019, to December 31, 2021, including detainees transported to offsite hospitals for emergency care; detainees in mental health observation; suicide watches, constant watch, and mental health observation; and suicide attempts (or self-harm).

### CA DOJ

The CA DOJ reviews ICE detention centers annually. To date, it has conducted reviews in 2019, 2021, and 2022.^16,17,18^ Age and sex (female, male, transgender, not reported) of individuals held in detention were available and obtained from CA DOJ 2019 and 2022 reports (eTable 1 in Supplement 1).

### EMS-Reported Emergencies

California detention centers that exclusively housed detained immigrants and were open for at least 2 years during the study period were eligible for inclusion. Five detention centers were identified: Adelanto ICE Processing Center, Golden State Annex, Imperial Regional Detention Facility, Mesa Verde ICE Processing Center, and Otay Mesa Detention Center. All detention centers housed both male and female immigrants. Local EMS regulatory authorities identified the agencies that respond to 911 activations at these facilities. Three of the 5 agencies responded to data requests. Deidentified 911 emergency responses were identified by local EMS agencies using facility addresses. Agency data were available from 2018 to 2022 for 2 centers; the third agency provided data beginning in 2019. Emergency responses were cleaned to remove EMS encounters for which the agency was not the primary care unit for the patient or had no patient contact. Nonprimary EMS responses were defined as (1) response to the same facility within 5 minutes of another EMS unit, (2) same age and sex of patient cared for by the primary unit, and (3) disposition categorized as transferred to another EMS unit. Information on race and ethnicity was not reported in the CA DOJ, DHS ICE, or EMS data.

### Statistical Analysis

A Poisson model was used in which EMS-reported emergency care was the outcome and demographics, facility, and their interaction were the exposure, using an offset to control for differences in census among sites based on CA DOJ reports. Post hoc comparisons of sex were made at each facility. Two-sided hypothesis testing and an a priori significance level of .05 were used. Of note, individuals identified as transgender or sex not reported (9 of 3557 [0.3%]) were excluded from the analysis given small sample sizes.

Emergencies reported by EMS were analyzed descriptively to assess vital signs, the primary symptom, EMS provider (emergency medical technician or paramedic) impression, and treatment provided. Data were reviewed for abnormal vital signs (defined as heart rate <60 or ≥100 beats per minute, systolic blood pressure <90 or ≥180 mm Hg, respiratory rate <12 or ≥24 breaths per minute, or oxygen saturation <90%).^19^ Extreme vital signs were reviewed. If incongruent with the clinical context, the vital sign was replaced with the patient’s median vital sign value for that measurement. If no other vital signs were available, data were noted as missing. Sixteen of 1224 (1.3%) patients were found to have at least 1 incongruent vital sign that was modified as described.

Primary symptoms and EMS provider impressions were reviewed. Similar entries were pooled (ie, chest wall pain and cardiac chest pain were both called chest pain). EMS provider impressions were stratified by sex to describe and compare the most frequent emergencies by sex, and the 10 most frequent impressions were reported. The total number of monthly psychiatric-related EMS-reported emergencies were compared with ICE-reported mental health observations, suicide watches, and suicide attempts by detention center. Medications and procedures were reviewed and duplicates merged.

Emergencies reported by EMS were analyzed to assess the absolute number per month per center. The number of emergencies was divided by the facility census to calculate the EMS-reported emergencies per 1000 population per month.^12^ Complete population data were restricted to July 1, 2018, through December 31, 2022. For months with missing population data (18 of 153 [11.8%]), values were imputed by taking the mean population of the month preceding and succeeding the missing data. Total monthly EMS-reported emergencies were compared with ICE-reported emergencies. Descriptive and statistical analyses were performed using R, version 4.2.2 software (R Foundation for Statistical Computing).

## Results

**Figure 1.**
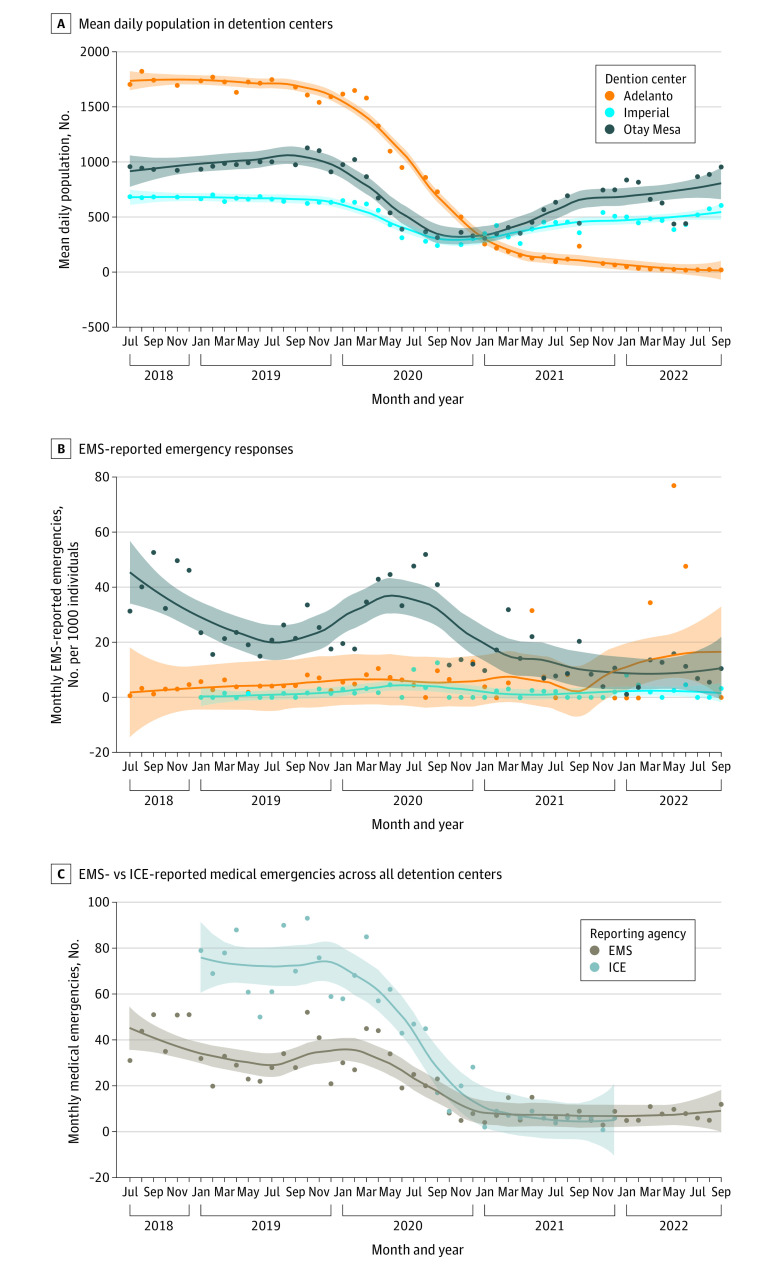
Detention Center Time Series Trends Monthly point values with locally estimated scatterplot smoothing lines (span of 0.5). Shaded areas indicate 95% CIs. EMS indicates emergency medical services; ICE, US Immigration and Customs Enforcement.

The 3 centers were run by private corporations, including CoreCivic, The GEO Group, and Management and Training Corporation (Table 1). The mean daily population at the 3 centers was 775 individuals per center (range, 504 [Imperial Regional Detention Facility] to 1001 [Adelanto ICE Processing Center]). In March 2020, concurrent with the onset of the COVID-19 pandemic, the mean daily population at all 3 detention centers decreased dramatically (Figure 1A). At Imperial and Otay Mesa, the mean daily population returned to prepandemic numbers by August 2022. All detention centers had medical services and personnel onsite provided by private corporations.

**Table 1.** Overview of California US Immigration and Customs Enforcement (ICE) Detention Centers That Exclusively House Detained Immigrants

Detention center	Time range		City	County	Facility type	Facility operator	Mean daily population	Medical personnel onsite	No. of medical personnel^a^	Medical organization
	EMS-reported data	ICE-reported data								
Adelanto ICE Processing Center	Jan 1, 2018, to Dec 31, 2022	Jul 1, 2018, to Sep 30, 2022	Adelanto	San Bernadino	DIGSA (2018-2020); CDF (2021-2022)	GEO	1001	Yes	90	Wellpath^b^
Imperial Regional Detention Facility	Jan 1, 2019, to Dec 31, 2022	Jul 1, 2018, to Sep 30, 2022	Calexico	Imperial	DIGSA (2018-2019); CDF (2020-2022)	M&TC	504	Yes	35	M&TC
Otay Mesa Detention Center	Jan 1, 2018, to Dec 31, 2022	Jul 1, 2018, to Sep 30, 2022	San Diego	San Diego	CDF	CoreCivic^c^	761	Yes	26	IHSC (2018-2020) CoreCivic (2021)
Golden State Annex^d^	NA	Dec 1, 2020, to Sep 30, 2022	McFarland	Kern	CDF	GEO	111	Yes	55	NA
Mesa Verde ICE Processing Center^d^	NA	Jul 1, 2018, to Sep 30, 2022	Bakersfield	Kern	DIGSA (2018); CDF (2019-2022)	GEO	219	Yes	27	GEO

Abbreviations: CDF, contracted detention facility; DIGSA, Dedicated Intergovernmental Service Agreement; EMS, emergency medical services; GEO, The GEO Group; IHSC, ICE Health Service Corps; M&TC, Management and Training Corporation; NA, not applicable.

^a^ Mean reported medical personnel obtained from ICE end-of-fiscal year reports.

^b^ Formerly known as Correct Care Solutions.

^c^ Formerly Corrections Corporation of America.

^d^ These EMS agencies did not respond to data requests; centers not included in study analysis.

From January 1, 2018, to December 31, 2022, there were 1224 EMS-reported emergencies across the 3 detention centers (median, 68 [IQR, 10-88] emergencies per center per month). The majority of individuals were male (881 [72%] vs female, 338 [28%]) with a median age of 39.0 (IQR, 30.0-49.0) years (Table 2). When stratified by center, the mean (SD) population age for individuals in detention ranged from 29.3 (9.5) to 42.8 (8.4) years, whereas that for EMS-reported emergencies ranged from 32.5 (9.4) to 44.8 (10.6) years (Table 2). The proportion of female individuals in detention was similar across centers (Adelanto, 114 of 881 [13%]; Imperial, 67 of 628 [11%]; Otay Mesa, 114 of 840 [14%]). At Adelanto and Imperial, the rate of EMS-reported emergencies was less for females than for males (female-to-male rate ratio: Adelanto, 0.51 [95% CI, 0.31-0.87]; Imperial, 0.86 [95% CI, 0.31-2.43]), whereas at Otay Mesa, the rate of EMS-reported emergencies was higher for females than for males (female-to-male rate ratio, 1.60; 95% CI, 1.40-183) (Table 2; eTables 2 and 3 in Supplement 1). The female-to-male rate ratio for EMS-reported emergencies at Otay Mesa was significantly different than that at Adelanto (rate ratio, 3.11; 95% CI, 1.88-5.57).

**Table 2.** Demographics of Emergency Medical Services (EMS)–Reported Emergencies (January 1, 2018, to December 31, 2022) vs Mean Detention Center Population (Approximately 2015-2021)

Characteristic	Adelanto ICE Processing Center		Imperial Regional Detention Facility		Otay Mesa Detention Center		Total	
	Female^a^	Male^a^	Female^a^	Male^a^	Female^a^	Male^a^	Female^a^	Male^a^
**Sex**								
Detention center–reported No. of total No. (%)^b^	114 of 881 (13)	767 of 881 (87)	67 of 628 (11)	561 of 628 (89)	114 of 840 (14)	726 of 840 (86)	295 of 2349 (13)	2054 of 2349 (87)
EMS-reported emergency								
No. of total No. (%)^c^	15 of 239 (6)	224 of 239 (94)	4 of 43 (9)	39 of 43 (91)	319 of 937 (34)	618 of 937 (66)	338 of 1219 (28)	881 of 1219 (72)
Female-to-male rate ratio (95% CI)^d^	0.51 (0.31-0.87)		0.86 (0.31-2.43)		1.60 (1.40-1.83)		Not applicable	
**Age, y**								
Detention center–reported								
Mean (SD)^e^	42.8 (8.4)	41.1 (11.8)	29.3 (9.5)	31.4 (8.8)	29.4 (9.0)	31.9 (8.9)	NA	NA
No. of individuals	9	70	70	506	114	701	NA	NA
EMS-reported emergency^f^								
Mean (SD)	41.9 (12.6)	41.4 (11.8)	44.8 (10.6)	32.5 (9.4)	37.3 (13.0)	41.7 (12.9)	37.61 (12.9)	41.2 (12.6)
Median (IQR)	45.0 (34.0-48.0)	42.0 (32.0-49.0)	41.0 (37.8-48.0)	31.0 (25.0-37.5)	35.0 (27.0-47.0)	40.0 (32.0-50.0)	36.0 (27.0-47.0)	40.0 (32.0-49.0)
No. of individuals	15	223	4	39	319	618	338	880

Abbreviation: NA, not available.

^a^ California Department of Justice (CA DOJ) reports and EMS agencies did not clarify how sex vs gender was obtained; we have assumed this variable to be representative of sex.

^b^ Detention population sex based on mean of 2019 and 2022 CA DOJ report. eTable 1 in Supplement 1 provides the full data range by center. The CA DOJ reports mentioned 4 individuals who identified as transgender. These individuals were excluded from the analysis given the small sample size.

^c^ The EMS data included 3 individuals whose sex was reported as other and 2 individuals with unknown sex. These individuals were excluded from the analysis given the small sample size.

^d^ Based on Poisson model regression of EMS-reported emergencies for female vs women with population demographics as offset (eTables 2 and 3 in Supplement 1).

^e^ Detention population age based on 2022 CA DOJ report. eTable 1 in Supplement 1 provides the full data range by center. Data on median age and total population age were NA.

^f^ Age not reported for 1 individual.

Almost one-third of patients had at least 1 abnormal vital sign during their EMS encounter (357 of 1220 [29.2%]) (Table 3). The most frequent abnormal vital sign was a heart rate greater than 100 beats per minute (162 of 1219 [13.3%]). Among 1224 individuals, the top 3 reported primary symptoms were chest pain (256 [20.9%]), abdominal pain (165 [13.5%]), and altered mental status (77 [6.3%]), while the top 3 EMS provider impressions were chest pain (244 [19.9%]), traumatic injury (134 [10.9%]), and abdominal pain (131 [10.7%]) (eTable 4 in Supplement 1). Respiratory distress necessitated EMS response for 47 individuals (3.8%). Three individuals (0.2%) were identified as having an ST elevation myocardial infarction, and 3 individuals (0.2%) experienced a cardiac arrest. Pregnancy-related emergencies accounted for 12.4% (42 of 338) of emergencies in females, the third most common type of emergency for females in detention (eTable 5 in Supplement 1).

**Table 3.** Emergency Medical Services–Reported Vital Signs

Vital sign	Mean (SD) or No. (%)
Total No. of patients	1224
Heart rate, beats per min (n = 1219)	
Patients’ median heart rate	83.6 (17.6)
No. of patients with any HR < 60	46 (3.8)
No. of patients with any HR ≥ 100	162 (13.3)
SBP, mm Hg (n = 1217)	
Patients’ median systolic blood pressure	139.9 (25.6)
No. of patients with any SBP < 90	12 (1.0)
No. of patients with any SBP ≥ 180	92 (7.5)
Respiratory rate, breaths per min (n = 1200)	
Patients’ median respiratory rate	18.0 (3.8)
No. of patients with any rate < 12	10 (0.8)
No. of patients with any rate ≥ 24	74 (6.0)
Oxygen saturation, % (n = 1177)	
Patient’s median oxygen saturation	98.0 (2.5)
No. of patients with any saturation < 90	10 (0.8)
Any abnormal vital sign (n = 1220)	357 (29.2)

Abbreviation: SBP, systolic blood pressure.

**Figure 2.**
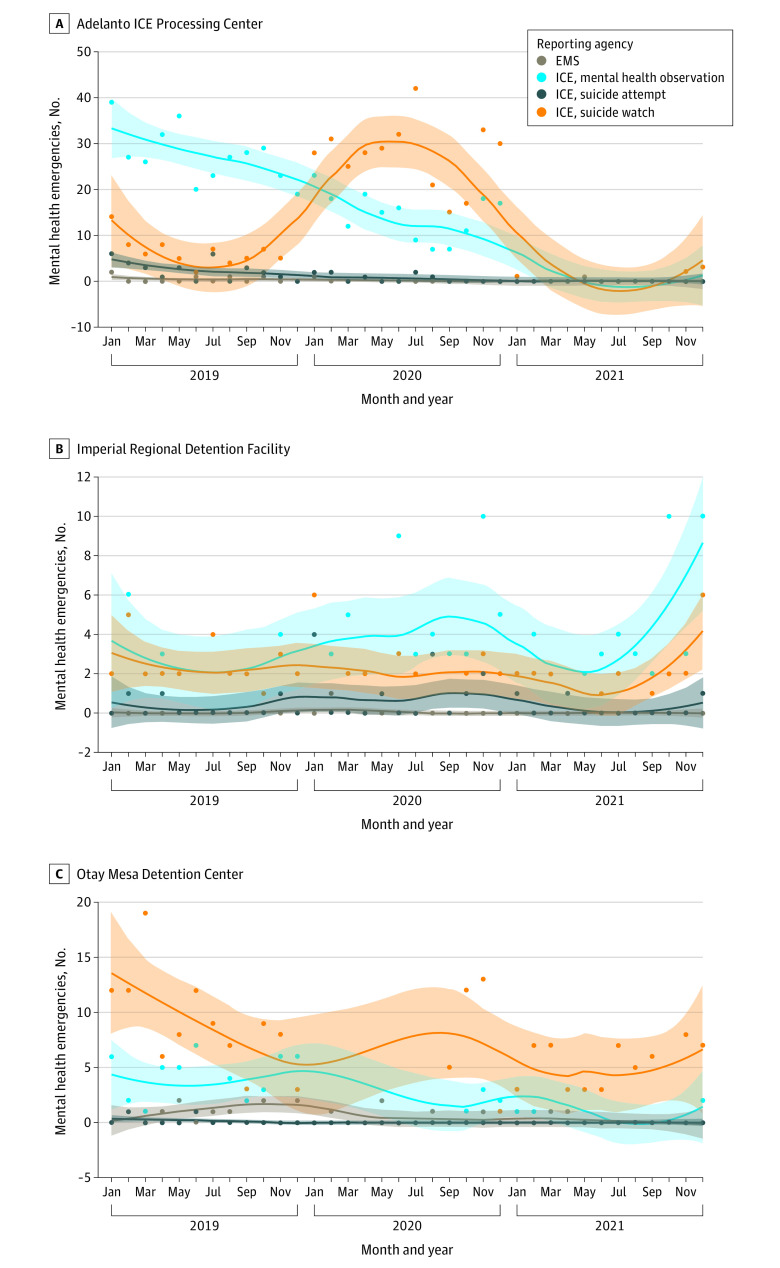
Emergency Medical Services (EMS)–Reported Mental Health Emergencies vs US Immigration and Customs Enforcement (ICE)–Reported Mental Health Encounters Monthly point values with locally estimated scatterplot smoothing lines (span of 0.5). Shaded areas indicate 95% CI.

Approximately 4% of EMS-reported emergencies were related to psychiatric or behavioral concerns (48 of 1219 [3.9%]). The mean (SD) number of EMS-reported psychiatric emergencies per month per center was 0.52 (1.13) (Figure 2). In comparison, the mean (SD) monthly ICE-reported mental health encounters per center were 7.13 (9.38) for mental health observations, 6.91 (8.57) for suicide watches, and 0.53 (1.26) for suicide attempts (Figure 2).

Among 1224 patients, the most common EMS-administered medications were nitroglycerin (175 [14.3%]), aspirin (158 [12.9%]), and normal saline (102 [8.3%]) (eTable 6 in Supplement 1). Commonly performed procedures included intravenous line placement (497 [40.6%]), electrocardiogram (276 [22.5%]), and blood glucose measurement (175 [14.3%]) (eTable 6 in Supplement 1).

The monthly rate of EMS-reported emergencies increased at Otay Mesa Detention Center from March to September 2020, with a peak of 52 emergencies per 1000 detained immigrants in August 2020 (Figure 1B). Imperial Regional Detention Facility showed an increase in emergencies from July to September 2020, with a peak of 12 emergencies per 1000 detained immigrants in September 2020 (Figure 1B). Adelanto ICE Processing Center showed an intermittent increase in the rate of EMS-reported emergencies in the springs of 2021 and 2022, with a peak of 77 emergencies per 1000 detained immigrants in May 2022 (Figure 1B).

From January 2019 to December 2021, there were 742 EMS-reported emergencies, compared with 1481 ICE-reported medical emergencies. The median number of monthly EMS-reported emergencies across all 3 centers was 3 (IQR, 0-9), while the median number of monthly ICE-reported emergencies was 4 (IQR, 1-15). The number of ICE-reported medical emergencies was higher across all 3 detention centers from January 2019 through July 2020 (Figure 1C).

## Discussion

The findings of this study expand our understanding of medical emergencies that occur in ICE detention facilities. Prior research has shown that deaths in detention facilities occur in predominantly young, male individuals.^4,5,6^ We found that EMS-reported medical emergencies were disproportionately for females at the Otay Mesa Detention Center, with 12% of all EMS-reported emergencies for female patients due to pregnancy concerns. The findings are particularly salient given the recent ICE directive 11032.4, effective July 1, 2021, that mandated that “ICE should not detain, arrest, or take into custody for an administrative violation of the immigration laws individuals known to be pregnant, postpartum, or nursing” and that officials should ensure “expeditious release, where legally authorized, of individuals known to be pregnant, postpartum, or nursing already detained in ICE custody.”^20^ Seven EMS-reported emergencies for pregnancy-related concerns occurred after July 1, 2021, indicating that Otay Mesa continued to house pregnant individuals despite ICE directives. It is unclear whether this higher rate of emergency activations at Otay Mesa represents higher rates of illness vs higher rates of monitoring and use of emergency services for females in detainment. California DOJ inspections documented that all 3 facilities included in this study “impermissibly house female detainees in restrictive housing under conditions disparate of those of male detainees.”^17^^(p26)^ Historically, women in ICE detention have experienced medical maltreatment, including unnecessary gynecologic procedures^21^ and reports of sexual assault.^22^

The findings also show a low percentage of EMS-reported emergencies for mental health crises. On average, the number of EMS-reported responses for psychiatric emergencies were 0.52 per month. In comparison, the average number of ICE-reported mental health observations were 7.13 per month, while the number of suicide attempts was 0.53 per month. The data might be interpreted as appropriate management of psychiatric crises in-house by medical staff at detention centers, with activation only when an individual has attempted suicide. However, the CA DOJ reports highlighted severe deficiencies in staffing of mental health professionals, delays in access to mental health care, dangerous use of solitary confinement, and lack of appropriate prevention of suicide harms in all 3 detention centers.^16,17^ Suicide accounted for 14.5% of deaths in ICE detention centers from 2011 to 2018^4^ and 25.7% from 2018 to 2020.^5^ According to 1 review of deaths from 2010 to 2020, the rate of suicide in ICE detention centers increased by 11-fold in 2020 compared with the prior 10-year average.^23^ Additional research is needed to better understand the reason for the relatively low rates of 911 activation for psychiatric crises seen in this study.

The temporal data presented here suggest that the rate of medical emergencies at detention facilities increased with the onset of the COVID-19 pandemic according to both EMS- and ICE-reported data. This increase in emergencies occurred despite efforts to reduce capacity at facilities, including ICE guidelines to reduce capacity to 75% and several court-ordered mandates, such as the release of approximately 50 detained immigrants at Otay Mesa in May 2020 and 250 detained immigrants at Adelanto in October 2020.^18,24,25,26,27^

Approximately 30% of individuals had at least 1 abnormal vital sign during their EMS encounter. A review of deaths in ICE custody showed an association between abnormal vital signs and critical illness resulting in death, suggesting a need to evaluate care delivered by ICE medical staff as well as follow-up of outcomes for individuals who had abnormal vital signs during their EMS encounter.^4^

During the 2019 to 2021 reporting period, there were 742 EMS-reported emergencies compared with 1481 ICE-reported medical emergencies. This discrepancy suggests that the EMS data are an underestimation of medical emergencies that occur in ICE detention centers. ICE provided no additional data to clarify the etiology of these emergencies or why 911 was not activated. As has been documented in death reviews, ICE may be transporting patients to emergency departments by private vehicle^6^; if so, one-half of patients would have been transported privately, raising questions about why this practice is common. ICE is also known for errors in provided data.^28^ Without additional information, it is challenging to interpret ICE-reported emergencies. Finally, although responding EMS agencies were confirmed with regional leadership, it is possible that other agencies may be responsible for 911 responses or that detention centers were listed under a different address.

### Limitations

This study has some limitations. While the study was limited to 3 ICE detention centers in California, their private operators represent 3 of the 4 predominant for-profit prison companies that contract with ICE in a landscape where more than 90% of detained immigrants are housed in facilities run by private corporations.^29,30,31,32^ The 2 remaining detention centers in California that were eligible but not included were also managed by The GEO Group and represent less than 15% of the eligible study population. While operational procedures are not publicly available, there may be similarities in protocols at facilities elsewhere in the country that are operated by 1 of the private companies in this study. Given stricter oversight in California, the data described here may represent higher standards of medical care than elsewhere.

The study data were limited to detention centers that exclusively house detained immigrants. As of 2022, more than 80% of individuals in ICE detention were detained in a facility that exclusively houses immigrants.^12^ In California, there was only 1 additional eligible facility that did not house detained immigrants exclusively. This facility closed in early 2023,^33^ and during its final years, it housed, on average, fewer than 10 detainees.^12^

Additional limitations regarding the EMS data include the possibility that a 911 emergency response at a detention center may have been for an employee or visitor rather than a detained immigrant. Upon review of 911 dispatch calls at a detention center in Georgia, less than 4% of 911 dispatches were for an employee or visitor.^34^ Given that the EMS-reported emergencies are less, rather than more, than ICE-reported medical emergencies, employee and visitor medical emergencies may not entail a large proportion of the data presented here.

Finally, EMS-reported emergencies only occur when detention staff call 911, as detained individuals do not have autonomy to activate 911 themselves. Death reviews have shown that requests for 911 response are sometimes delayed by senior medical personnel.^6^

## Conclusions

This cross-sectional study represents a first look at medical emergencies at 3 ICE detention centers in California, an environment in which detention systems face heightened scrutiny and restrictions compared with many states elsewhere in the country.^9,10,33^ Initial data suggest concerns regarding care for vulnerable populations, including pregnant women and individuals with psychiatric illness. Furthermore, the study findings highlight the difficulty in obtaining and interpreting data. Attempts to better understand emergency care in detention centers required use of multiple sources to obtain basic information and showed discrepancies in EMS- vs ICE-reported data. Increased transparency of how medical emergencies are managed and when 911 is activated in ICE detention centers is required to ensure that this publicly funded health care system meets the needs of individuals in detention.

## Notes

Supplement 1. **eTable 1.** Date Span of Data Provided by Facilities in California Department of Justice (CA DOJ) Reports **eTable 2.** Poisson Regression Model of EMS-Reported Emergencies by Sex and Facility **eTable 3.** Female-to-Male Rate Ratio of EMS-Reported Emergencies by Detention Center **eTable 4.** EMS Primary Symptoms and Provider Impressions **eTable 5.** EMS Most Common Provider Impressions by Sex **eTable 6.** EMS Interventions

Supplement 2.
Data Sharing Statement
