# Supplementary material for: Emergency Medical Responses at US Immigration and Customs Enforcement Detention Centers in California
Source: JAMA Netw Open. 2023 Nov 29;6(11):e2345540. doi: 10.1001/jamanetworkopen.2023.45540 (PMC10687658; doi:10.1001/jamanetworkopen.2023.45540)
Supplement: Supplement 1. — eTable 1. Date Span of Data Provided by Facilities in California Department of Justice (CA DOJ) Reports eTable 2. Poisson Regression Model of EMS-Reported Emergencies by Sex and Facility eTable 3. Female-to-Male Rate Ratio of EMS-Reported Emergencies by Detention Center eTable 4. EMS Primary Symptoms and Provider Impressions eTable 5. EMS Most Common Provider Impressions by Sex eTable 6. EMS Interventions [file jamanetwopen-e2345540-s001.pdf]

Supplemental Online Content

Dekker AM, Farah J, Parmar P, Uner AB, Schriger DL. Emergency medical responses at US Immigration and Customs Enforcement detention centers in California. *JAMA Netw Open*. 2023;6(11):e2345540. doi:10.1001/jamanetworkopen.2023.45540

eTable 1. Date Span of Data Provided by Facilities in California Department of Justice (CA DOJ) Reports

eTable 2. Poisson Regression Model of EMS-Reported Emergencies by Sex and Facility

eTable 3. Female-to-Male Rate Ratio of EMS-Reported Emergencies by Detention Center

eTable 4. EMS Primary Symptoms and Provider Impressions

eTable 5. EMS Most Common Provider Impressions by Sex

eTable 6. EMS Interventions

This supplemental material has been provided by the authors to give readers additional information about their work.

© 2023 Dekker AM et al. *JAMA Network Open.*

eTable 1. Date Span of Data Provided by Facilities in California Department of Justice (CA DOJ) Reports

	Age	Sex[a]	
**Detention Center**	**CA DOJ 2022**	**CA DOJ 2019**	**CA DOJ 2022**
Adelanto ICE Processing Center	November 15, 2021	June 2017 to October 2018	November 15, 2021
Imperial Regional Detention Facility	October 5, 2021	July 2017 to November 2018	October 5, 2021
Otay Mesa Detention Center	October 4, 2021	October 2015 to October 2018	October 4, 2021

[a]CA DOJ reports did not clarify how sex/gender demographic was obtained; the authors have assumed this variable to be representative of sex.

eTable 2. Poisson Regression Model of EMS-Reported Emergencies by Sex and Facility

	Estimate Coefficient	Exponentiated Coefficients	95% CI for Estimate	*p*-value
**Intercept**	-1.487	0.23	0.20, 0.26	<0.001
**Imperial**	-1.246	0.29	0.20, 0.40	<0.001
**Otay Mesa**	0.710	2.03	1.75, 2.37	<0.001
**Female**	-0.665	0.51	0.29, 0.87	0.01
**Imperial*Female**	0.522	1.69	0.46, 4.93	0.38
**Otay Mesa*Female**	1.136	3.11	1.88, 5.57	<0.001

eTable 3. Female-to-Male Rate Ratio of EMS-Reported Emergencies by Detention Center

	F:M Rate Ratio	95% CI	*p*-value
**Adelanto ICE Processing Center**	0.51	(0.31, 0.87)	0.01
**Imperial Regional Detention Facility**	0.86	(0.31, 2.43)	0.79
**Otay Mesa Detention Center**	1.60	(1.40, 1.83)	<0.001

© 2023 Dekker AM et al. *JAMA Network Open.*

eTable 4. EMS Primary Symptoms and Provider Impressions

**Primary Symptom,** No. (%) (exclude n<10)	**Individuals (N=1224)**
Chest pain	256 (20.9)
Abdominal pain	165 (13.5)
Altered mental status	77 (6.3)
Weakness	68 (5.6)
Headache	61 (5.0)
Seizure	49 (4.0)
Extremity pain	43 (3.5)
Shortness of breath	43 (3.5)
Back pain	37 (3.0)
Dizziness	36 (2.9)
HEENT pain	32 (2.6)
Nausea/vomiting	28 (2.3)
Abnormal behavior	27 (2.2)
Neck pain	22 (1.8)
Syncope/collapse	21 (1.7)
Anxiety	20 (1.6)
Bleeding / Hemorrhage	19 (1.6)
Pelvic / Perineal pain	14 (1.1)
Cough	11 (0.9)
Vaginal bleeding	11 (0.9)
**Provider Impression,** No. (%) (exclude n<10)	**Individuals (N=1224)**
Chest pain	244 (19.9)
Traumatic injury	134 (10.9)
Abdominal pain	131 (10.7)
Seizure	80 (6.5)
Weakness	72 (5.9)
Behavioral / Psychiatric crisis	48 (3.9)
Respiratory distress	47 (3.8)
Non-traumatic body pain	42 (3.4)
Pregnancy complication / labor	42 (3.4)
Syncope / Near syncope	36 (2.9)
Altered LOC, no hypoglycemia or seizure	33 (2.7)
Overdose / Poisoning / Ingestion	32 (2.6)
Headache, non-traumatic	28 (2.3)
Dizziness / Vertigo	24 (2.0)
Stroke / CVA / TIA	24 (2.0)
Hypertension	22 (1.8)
Pain / Swelling in extremity, non-traumatic	18 (1.5)
Allergic reaction	15 (1.2)
Cardiac dysrhythmia	12 (1.0)
Nausea/vomiting	12 (1.0)
Hyperglycemia	10 (0.8)
Vaginal bleeding	10 (0.8)

© 2023 Dekker AM et al. *JAMA Network Open.*

eTable 5. EMS Most Common Provider Impressions by Sex

Female		Male	
**Provider Impression**	**n (%), N=338**	**Provider Impression**	**n(%), N=881**
1. Abdominal pain	56 (16.6%)	1. Chest pain	199 (22.6%)
2. Chest pain	45 (13.3%)	2. Traumatic injury	104 (11.8%)
3. Pregnancy complication/labor	42 (12.4%)	3. Abdominal pain	75 (8.5%)
4. Traumatic injury	29 (8.6%)	4. Seizure	58 (6.6%)
5. Seizure	22 (6.5%)	5. Weakness	52 (5.9%)
6. Weakness	20 (5.9%)	6. Behavioral/psychiatric crisis	41 (4.7%)
7. Syncope/near-syncope	12 (3.6%)	7. Respiratory distress	40 (4.5%)
8. Non-traumatic body pain	11 (3.3%)	8. Non-traumatic body pain	31 (3.5%)
9. Stroke/CVA/TIA	10 (3.0%)	9. Overdose/poisoning/ingestion	28 (3.2%)
10. Headache, non-traumatic	9 (2.7%)	10. Altered LOC, no hypoglycemia or seizure	26 (3.0%)

eTable 6. EMS Interventions

**Medication,** No. (%)	**Individuals (N=1224)**
Nitroglycerin	175 (14.3)
Aspirin	158 (12.9)
Normal saline	102 (8.3)
Ondansetron	92 (7.5)
Oxygen	55 (4.5)
Albuterol/Ipratropium	30 (2.5)
Fentanyl	28 (2.3)
Acetaminophen	24 (2.0)
Midazolam	24 (2.0)
Diphenhydramine	10 (0.8)
Glucose/Dextrose	9 (0.7)
Naloxone	8 (0.7)
Adenosine	4 (0.3)
Atropine	3 (0.2)
Ketamine	3 (0.2)
Activated charcoal	2 (0.2)
Epinephrine 1:1,000	2 (0.2)
Lidocaine	2 (0.2)
Amiodarone	1 (0.1)
Epi 1:10,000	1 (0.1)
Epi 1:100,000	1 (0.1)
Glucagon	1 (0.1)
**Procedure,** No. (%)	**Individuals (N=1224)**
IV placement	497 (40.6)
Electrocardiogram	276 (22.5)
Blood glucose	175 (14.3)
Spinal immobilization	52 (4.2)
Active external cooling	3 (0.2)
IO placement	3 (0.2)
Wound care	3 (0.2)
Splint application	2 (0.2)
Bag valve mask ventilation	2 (0.2)
Nasopharyngeal airway	2 (0.2)
Vagal stimulation	2 (0.2)
Airway suction	1 (0.1)

© 2023 Dekker AM et al. *JAMA Network Open.*
