# Supplementary material for: Emergency Medical Responses at US Immigration and Customs Enforcement Detention Centers in California
Source: JAMA Netw Open. 2023 Nov 29;6(11):e2345540. doi: 10.1001/jamanetworkopen.2023.45540 (PMC10687658; doi:10.1001/jamanetworkopen.2023.45540)
Supplement: Supplement 2. — Data Sharing Statement [file jamanetwopen-e2345540-s002.pdf]

Data Sharing Statement

Dekker. Emergency Medical Responses at US Immigration and Customs Enforcement Detention Centers in California. *JAMA Netw Open*. Published November 29, 2023. doi:10.1001/jamanetworkopen.2023.45540

Data
Data available: No
